# Serelaxin Treatment Reduces Oxidative Stress and Increases Aldehyde Dehydrogenase-2 to Attenuate Nitrate Tolerance

**DOI:** 10.3389/fphar.2017.00141

**Published:** 2017-03-21

**Authors:** Chen Huei Leo, Dhanushke T. Fernando, Lillie Tran, Hooi Hooi Ng, Sarah A. Marshall, Laura J. Parry

**Affiliations:** School of BioSciences, The University of Melbourne, ParkvilleVIC, Australia

**Keywords:** serelaxin, aldehyde dehydrogenase-2, aorta, nitrates, oxidative stress

## Abstract

**Background:** Glyceryl trinitrate (GTN) is a commonly prescribed treatment for acute heart failure patients. However, prolonged GTN treatment induces tolerance, largely due to increased oxidative stress and reduced aldehyde dehydrogenase-2 (ALDH-2) expression. Serelaxin has several vasoprotective properties, which include reducing oxidative stress and augmenting endothelial function. We therefore tested the hypothesis in rodents that serelaxin treatment could attenuate low-dose GTN-induced tolerance.

**Methods and Results:** Co-incubation of mouse aortic rings *ex vivo* with GTN (10 μM) and serelaxin (10 nM) for 1 h, restored GTN responses, suggesting that serelaxin prevented the development of GTN tolerance. Male Wistar rats were subcutaneously infused with ethanol (control), low-dose GTN+placebo or low-dose GTN+serelaxin via osmotic minipumps for 3 days. Aortic vascular function and superoxide levels were assessed using wire myography and lucigenin-enhanced chemiluminescence assay respectively. Changes in aortic ALDH-2 expression were measured by qPCR and Western blot respectively. GTN+placebo infusion significantly increased superoxide levels, decreased ALDH-2 and attenuated GTN-mediated vascular relaxation. Serelaxin co-treatment with GTN significantly enhanced GTN-mediated vascular relaxation, reduced superoxide levels and increased ALDH-2 expression compared to GTN+placebo-treated rats.

**Conclusion:** Our data demonstrate that a combination of serelaxin treatment with low dose GTN attenuates the development of GTN-induced tolerance by reducing superoxide production and increasing ALDH-2 expression in the rat aorta. We suggest that serelaxin may improve nitrate efficacy in a clinical setting.

## Introduction

Organic nitrates such as nitroglycerin (GTN) are widely used for the acute treatment of heart diseases including chronic congestive heart failure, coronary artery disease and acute heart failure (AHF) (Daiber and Münzel, 2015). The beneficial effects of nitrates are mainly associated with their ability to dilate venous capacitance vessels, coronary arteries and the aorta, thereby improving left ventricular function and reducing myocardial workload (Abrams, 1995). The mechanisms underlying this vasodilation involve release of NO (and the metabolite 1,2 glyceryl dinitrite) in response to intracellular biotransformation of GTN by the ALDH-2 enzyme (Chen et al., 2002). This results in activation of soluble guanylate cyclase (sGC), leading to increased cyclic guanosine-3′,-5′-monophosphate (cGMP) levels and relaxation of the vascular smooth muscle (Münzel et al., 2005). GTN-dependent vasodilation of isolated aortic rings is reduced by daidzin, a highly specific inhibitor of ALDH-2 (Sydow et al., 2004). Moreover, mice deficient in mitochondrial ALDH-2 show impaired relaxation specifically to GTN, but not other nitrovasodilators such as sodium nitroprusside in the aorta (Chen et al., 2002).

Long-term continuous administration of organic nitrates has its limitations because of the rapid development of tolerance, cross tolerance to other nitrovasodilators, and endothelial dysfunction (Münzel et al., 2005, 2011). Patients treated with clinically relevant, low-dose GTN for 48 h develop tolerance to GTN and endothelial dysfunction in arterial and venous vessels. This is associated with decreased vascular expression of ALDH-2 and activity, and increased ROS production (Schulz et al., 2002; Hink et al., 2007). Interestingly, higher doses of GTN are routinely used to induce GTN tolerance and endothelial dysfunction in rodent vessels (Mülsch et al., 2001; Sydow et al., 2004; Wenzel et al., 2008). The effects of clinically relevant, low-dose GTN treatment on rodent vessels are less well-studied, particularly the development of tolerance and/or endothelial dysfunction. The mechanisms that lead to nitrate tolerance involve multiple factors, including impaired biotransformation of organic nitrates to NO, desensitization of sGC receptors, increased activity of phosphodiesterase, and increased vascular superoxide production (Münzel et al., 2005, 2011). This latter feature is a major limitation for the clinical use of GTN because oxidative stress induction is a characteristic of cardiovascular disease and actively contributes to its progression.

The recombinant form of human relaxin-2 (serelaxin) has emerged as a potential drug with long-term therapeutic advantages for patients with AHF (Teerlink et al., 2013). Follow-up studies in AHF patients reported that intravenous infusion of serelaxin rapidly reduced systemic vascular resistance (Ponikowski et al., 2014), an effect likely to be mediated through a vasodilatory action of serelaxin on systemic arteries as demonstrated in many animal studies and humans (Jeyabalan et al., 2003; Conrad, 2010; McGuane et al., 2011b; Leo et al., 2016a). The vascular actions of serelaxin occur through activation of the RXFP1, localized within the endothelium and smooth muscle cells of several arteries and veins, including the aorta (Jelinic et al., 2014; Ng et al., 2015). Serelaxin treatment in rats enhances endothelium-dependent relaxation via a combination of NO, EDH, and prostacyclin (PGI_2_). The contribution of these vasodilator pathways is influenced by duration of serelaxin treatment and varies between vascular beds (Jelinic et al., 2014; Leo et al., 2014b, 2016a,b). Under conditions of vascular dysfunction, serelaxin also has vasoprotective functions. Incubation of rat aortic rings with tumor necrosis factor-α *ex vivo* increased ROS production and impaired ACh-induced relaxation. Co-incubation of these aortic rings with serelaxin caused PI3K-dependent eNOS dephosphorylation at Thr^495^, eNOS phosphorylation at Ser^1177^ and Ser^633^, attenuated arginase II expression (Dschietzig et al., 2012), increased eNOS activity and improved ACh-mediated endothelium-dependent relaxation. Serelaxin treatment also reduced superoxide and nitrotyrosine levels (Dschietzig et al., 2012). Similarly, serelaxin treatment prevented aorta endothelial dysfunction under conditions of acute hyperglycemia (Ng et al., 2016) and chronic exposure to cigarette smoke (Pini et al., 2016). Prevention of endothelial dysfunction was partly explained by the reduction of oxidative stress and an upregulation of eNOS and PGI_2_ activity (Ng et al., 2016; Pini et al., 2016). Therefore, a key mechanism of serelaxin action is to maintain vascular function by reducing oxidative stress.

Interestingly, ancillary observations made during a clinical study in which patients with peripheral arterial disease were treated with relaxin intramuscularly for at least 3 months were suggestive of improvements in their nitroglycerin requirements. 3/20 patients with prior myocardial infarction and active angina were treated with nitroglycerin. Interestingly, all of these patients reported that their reliance on nitroglycerin was declined when relaxin was co-administered (Casten et al., 1960; Raleigh et al., 2016). Thus, serelaxin could perform two important functions in the vasculature of AHF patients who may require acute GTN therapy: (i) synergistic action with GTN to reduce systemic vascular resistance through a vasodilatory action, and (ii) prevent the development of tolerance to GTN (or other organic nitrates). Therefore, the aims of the present study were to investigate whether or not serelaxin attenuates GTN tolerance in the aorta using *ex vivo* studies in mice and *in vivo* experiments in rats. Our underlying hypothesis was that serelaxin attenuates GTN-induced overproduction of vascular ROS by increasing ALDH-2.

## Materials and Methods

All procedures were approved by the Faculty of Science Animal Experimentation Ethics Committee (The University of Melbourne, AEC1312780.1 and AEC1312840.1) and conformed to the National Health and Medical Research Council of Australia code of practice for the care and use of animals for scientific purposes.

### Animals

Male C57BL/6J mice (body weight 25–30 g) and Wistar rats (body weight 200–250 g) were purchased from the Animal Resource Centre (Canning Vale, WA, Australia). The animals were housed in the School of BioSciences Animal House Facilities (The University of Melbourne) in a 12L:12D cycle at 20°C, with standard food pellets (Barastock, Pakenham, VIC, Australia) and water provided *ad libitum*.

### Vascular Reactivity

Mice were killed via cervical dislocation under 2% isofluorane anesthesia and the aortae were isolated, cleared of fat and connective tissue, and cut into 1–2 mm long rings and mounted on a Mulvany–Halpern myograph (model 610 M, Danish Myo Technology, Aarhus, Denmark) using two 40 μm stainless steel wire. The output from the myograph was recorded and analyzed using a PowerLab data acquisition system and the program LabChart (ADInstruments, Bella Vista, NSW, Australia). The remainder of the aorta was snap frozen in liquid nitrogen and stored at -80°C for further analysis. After the aortic rings were mounted on the myograph, they were allowed to stabilize at zero tension for 15 min. After stabilization, the vessels were stretched to 5 mN during a normalization period of 45 min, and then maximally contracted using either high potassium saline solution (KPSS) or the thromboxane A_2_ mimetic, U46619 (1 μM). To confirm endothelium integrity, vessels were pre-contracted to 50% of the maximum KPSS response using phenylephrine (PE) (10–50 nM) or U46619 (10–50 nM), and then relaxed with the endothelium-dependent agonist ACh. The endothelium was considered to be functional if relaxation to ACh was >80% as described previously for the mouse (Ng et al., 2015) and rat aortae (Leo et al., 2016c). All experiments were performed at 37°C in the presence of 95% O_2_ and 5% CO_2_.

### *Ex vivo* Tolerance Experiments

The aortic rings were then incubated in either placebo (20 mM sodium acetate) or in the presence of GTN (1, 10, 30, or 100 μM), or serelaxin (1, 3, or 10 nM; kindly provided by Novartis Pharma AG, Switzerland) for a period of 60 min. Aortic rings were washed thoroughly every 15 min for 1 h after incubation. These concentrations of serelaxin were previously shown to result in vasodilation (McGuane et al., 2011a; Dschietzig et al., 2012; Ng et al., 2016). The vessels were pre-contracted to ∼50% of maximal contraction with the thromboxane A_2_ mimetic, U46619 (10–50 nM), and concentration-response curves to GTN (0.1 nM–10 μM) were obtained. At the end of the response curves, 10 μM of the endothelium-independent dilator levcromakalim (LVK) was added to induce maximum relaxation. To investigate if serelaxin treatment prevents tolerance to GTN *ex vivo*, the aortae were incubated with placebo alone or 10 μM of GTN in combination with serelaxin (3 or 10 nM) for 1-h, followed by a 1-h washout period. Subsequently, concentration-response curves to GTN (0.1 nM–10 μM) were evaluated in ∼50% pre-contracted aortae. Only one concentration response curve was performed on each aortic ring.

### *In vivo* Tolerance Model

Male Wistar rats were randomly divided into three groups: (i) controls (*n* = 14), (ii) GTN + placebo (*n* = 16), and (iii) GTN + serelaxin (*n* = 15). The rats were implanted with osmotic minipumps (Alzet Model 2001, Bioscientific, Gymea, NSW, Australia) to infuse low dose GTN (5 μg h^-1^) (Schulz et al., 2002; Hink et al., 2007) and/or serelaxin (4 μg h^-1^) (Leo et al., 2016c) subcutaneously under the back of the skin for 3 days. There was no control+serelaxin group because we have previously reported that serelaxin treatment for 3 days had no significant effects in the aorta under control conditions (Leo et al., 2016c). The control rats received placebo solution which was either ethanol or 20 mM sodium acetate. The dose of GTN and serelaxin were chosen to mimic clinically relevant concentrations in the treatment of acute coronary syndrome and acute myocardial infarction (Jugdutt and Warnica, 1989) and RELAX-AHF clinical trial (Teerlink et al., 2013) respectively.

Following 3 days of infusion, blood samples were obtained from the left ventricle via cardiac puncture under 2% isofluorane anesthesia. The rats were then killed by bilateral chest incision through the diaphragm and removal of the heart. Plasma concentrations of serelaxin (*n* = 8–10 per group) were measured in duplicate using the Human Relaxin-2 Quantikine ELISA Kit (R&D Systems, Minneapolis, MN, USA) following the manufacturer’s protocol. The limit of detection was 15.6 pg/ml and the intra- and inter coefficients of variation were 2.3–4.7 and 5.5–10.2%, respectively. The abdominal aorta was isolated and prepared for vascular reactivity experiments using the wire-myograph as described earlier. The aortic rings were pre-contracted to ∼50% of maximal contraction with phenylephrine, PE (1–10 μM) and concentration-response curves to GTN (0.1 nM–10 μM), the NO donor, diethylamine NONOate (DEA/NO, 0.1 nM–10 μM) and the endothelium-dependent vasodilator, ACh (1 nM–10 μM) were obtained. Only one concentration response curve to each vasodilator was performed on each aortic ring. To examine the basal NO activity, endothelium-intact aortic rings were sub-maximally contracted with PE (10–100 nM) to ∼20% of maximal contraction followed by the addition of the NOS inhibitor, _L_-NAME (200 μM). Under these conditions, a contractile response to _L_-NAME is considered to reflect the level of basal NOS activity (Leo et al., 2011; Kahlberg et al., 2016).

### Assessment of Reactive Oxygen Species

Superoxide anion production in the rat aortae was determined by lucigenin-enhanced chemiluminescence assay as previously described (Leo et al., 2012). Briefly, three 2 mm rings were cut and placed in wells containing modified Krebs- HEPES buffer (containing in mM 99.9 NaCl, 4.7 KCl, 1.2 MgSO_4_, 1 KH_2_PO_4_, 25 NaHCO_3_, 20 Na-HEPES, 11 glucose, and 2.5 CaCl_2_). To assess NADPH oxidase driven superoxide production, two rings were stimulated with NADPH (100 μM). One ring was exposed to the flavoprotein inhibitor that inhibits NADPH oxidase, diphenyliodonium (DPI, 5 μM). Tissue segments were first incubated with the superoxide dismutase (SOD) inhibitor DECTA (3 mM) for 45 min at 37°C and then washed for 2 min. They were then transferred to wells containing lucigenin (5 μM) and measured for luminescence in a FLUOstar Omega filter-based multimode microplate reader (BMG LABTECH, Melbourne, VIC, Australia). Superoxide production was calculated by subtracting the chemiluminescence signal obtained in blank wells from the signal detected in the well-containing the aortic ring and then normalized to dry tissue weight (mg).

### Western Blotting

Frozen endothelium-intact rat aortae were placed in a pre-chilled Wig-L-Bug^®^ capsule with a metal ball and pulverized with a Digital Wig-L-Bug^®^ amalgamator (Dentsply, Ltd, York, PA, USA). Protein extraction and western blot were performed as described previously (Leo et al., 2010). Briefly, samples were dissolved in 300 μL of ice-cold lysis buffer (100 mmol L^-1^ NaCl, 10 mmol L^-1^ Tris, 2 mmol L^-1^ EDTA, 0.5% w/v sodium deoxycholate, 1% vol/vol triton X-100, pH 7.4) with a protease and phosphatase inhibitor cocktail according to manufacturer’s instructions (Roche, Sydney, NSW, Australia). Total protein concentration of the samples was quantified using a BCA protein assay kit (ThermoScientific, Rockford, IL, USA). Equal amounts (20 μg) of protein homogenate were subjected to SDS-PAGE (10%) and Western blot analysis using rabbit primary antibodies (1:250, overnight, 4°C) against ALDH-2 (ABCAM, Cambridge, CB, UK). To normalize for the amount of protein, membranes were re-probed with actin (Sigma-Aldrich, St. Louis, MO, USA), which served as a loading control (1:2000). All proteins were detected by enhanced chemiluminescence (Amersham, GE Healthcare Life Sciences, Sydney, NSW, Australia) after incubation with HRP-conjugated secondary antibody (Millipore, Billerica, MA, USA) for 1 h at room temperature (1:2000). All protein bands were quantified by densitometry (Biorad Chemidoc, Sydney, NSW, Australia) and expressed as a ratio of the loading control.

### RNA Extraction and Quantitative PCR

Frozen rat aortae were pulverized as described previously (Leo et al., 2014a). Pulverized tissues were resuspended in 1 ml TriReagent (Ambion, Inc., Scoresby, VIC, Australia) and total RNA was then extracted according to the manufacturer’s instructions. RNA pellets were resuspended in 20 μl RNA Secure^TM^ (Ambion). Quality and quantity of RNA were determined using the NanoDrop ND1000 Spectrophotometer (Thermo Fisher Scientific Australia Pty Ltd, Scoresby, VIC, Australia) with A_260_:A_280_ ratios > 1.8 indicating sufficient quality for qPCR analysis. First strand cDNA synthesis used 1 μg of total RNA in a 20 μl reaction containing random hexamers (50 ng μL^-1^) and 200 units of Superscript^TM^ III (Invitrogen, Mulgrave, VIC, Australia). First-strand cDNA synthesis for all samples were performed simultaneously at 25°C for 10 min, 50°C for 50 min, and 85°C for 5 min. The comparative cycle threshold (2^-ΔCt^) method of qPCR was used to analyze *Aldh2*, eNOS (*Nos3*), *Dhfr*, and *Gch1* gene expression in the rat aortae. The latter two genes code for two enzymes that are involved in the regulation of the intracellular levels of the eNOS cofactor tetrahydrobiopterin (BH4), which preserves eNOS dimerization and improves endothelial function. Rat-specific forward/reverse primers and 6-carboxyl fluorescein-labeled (FAM) Taqman probes were designed and purchased from Biosearch Technologies (**Table 1**, Novato, CA, USA). Primers were designed to span intron/exon boundaries. qPCR was performed on the Applied Biosystems ViiA7 PCR machine (Life Technologies, Mulgrave, VIC, Australia) using 96-well plates with 10 μl volume reactions in triplicate containing SensiFAST^TM^ Probe Lo-Rox (BioRad, West Ryde, NSW, Australia) and 10 μmol L^-1^ of primers and FAM-labeled probe. Ribosomal 18S (*Rn18s*) was used as the reference gene. Negative template controls substituting cDNA with water or RT negative controls substituting the reverse transcriptase in the cDNA synthesis were included on each plate. For each sample, the mean *Rn18s* C_T_ triplicate value was subtracted from the mean gene of interest triplicate C_T_ value, and normalized to the reference gene. These normalized data (ΔCt) were then presented as a relative value (mean ± SEM).

**Table 1:** Forward and reverse primers of genes of interest.

Gene	Sequence	Accession ID
r18S FAM r18S Fwd r18S Rev	TGGAGCGATTTGTCTGGTTAATTCCGA GCATGGCCGTTCTTAGTTGG TGCCAGAGTCTCGTTCGTTA	NR_046237.1

rALDH2 FAM rALDH2 Fwd rALDH2 Rev	TGTGGTCAATATTGTTCCTGGA CACCGCTCACTGCACTCTAC AAGGCCACTTTGTCCACATC	NM_032416.1

rDHFR FAM rDHFR Fwd rDHFR Rev	GTAAAGTGGACATGGTCTGGGT TGCCAAAAGTCTGGATGATG CTGATTCATGGCTTCCTGGT	NM_130400.2

rGCH1 FAM rGCH1 Fwd rGCH1 Rev	ATGGTGATTGTGAAGGACATTG CACCAAGGGATACCAGGAGA AGGTGATGCTCACACATGGA	NM_024356.1

rNOS3 FAM rNOS3 Fwd rNOS3 Rev	CCGATACAACATACTTGAGG ATGAGTTCAGAGATTGGCATGA TTTCCACAGTGATGAGGTTGTC	NM_021838.2

*TaqMan probes were labeled with FAM. Sequences were acquired from the NCBI nucleotide database.*

### Reagents

All drugs were purchased from Sigma-Aldrich (St. Louis, MO, USA), except for U46619 and DEA/NO (Cayman Chemical, Ann Arbor, MI, USA), LVK (Tocris Chemicals, Bristol, UK) and GTN (Hospira, Melbourne, VIC, Australia). They were all dissolved in distilled water, with the exception of GTN and U46619, which were dissolved in 100% ethanol (final concentration less than 0.1% ethanol) as 1 mmol/l stock solution and subsequent dilutions were in distilled water. DEA/NO was dissolved in 0.1 M NaOH. Diphenyliodonium and LVK were dissolved in 100% DMSO (final concentration less than 0.1% DMSO).

### Statistical Analyses

All results are expressed as the mean ± standard error of the mean (SEM), n represents the number of animals per group. Concentration-response curves were computer fitted to a sigmoidal curve using non-linear regression (Prism version 5.0, GraphPad Software, USA) to calculate the sensitivity against each agonist (pEC_50_). Maximum relaxation (R_max_) to ACh, GTN, or DEA/NO was measured as a percentage of pre-contraction recorded at highest concentration. Statistical differences between group pEC_50_ and R_max_ values, superoxide levels and protein/gene expression were all compared via one-way ANOVA with *post hoc* Dunnett’s or Tukey’s tests where appropriate (Prism version 5.0, GraphPad Software, USA). *P* < 0.05 was considered statistically significant.

## Results

### Serelaxin Attenuates Development of Tolerance to GTN in the Mouse Aorta *Ex vivo*

**FIGURE 1:**
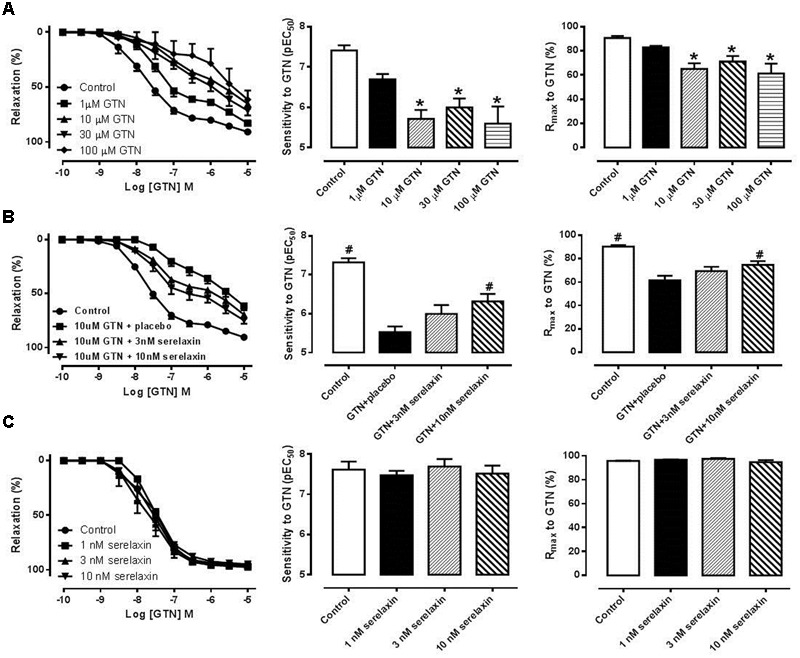
**(A)** Concentration-response curves, sensitivity (pEC50) and maximum relaxation (Rmax) to GTN after 1 h incubation of **(A)** either control or GTN (1, 10, 30, 100 μM), **(B)** control, 10 μM GTN+placebo, 10 μM GTN+3 nM serelaxin, 10 μM GTN+10 nM serelaxin, **(C)** either placebo or serelaxin (1, 3, 10 nM), followed by 1 h washout. Data are expressed as mean ± SEM, *n* = 5–8 per group. ^∗^*P* < 0.05 compared to control (one-way ANOVA, Dunnett’s test) #*P* < 0.05 vs. 10 uM GTN+placebo (one-way ANOVA, Dunnett’s test).

Incubation of mouse aortic rings with 10, 30, and 100 μM, but not 1 μM, of GTN for 1 h followed by a 1 h washout period caused a significant (*P* < 0.01) decrease in the sensitivity and maximum relaxation to GTN in a concentration-dependent manner (**Figure 1A**), indicating the development of tolerance to GTN *ex vivo*. Analysis of the area under the curve for GTN-mediated relaxation also produced similar findings (data not shown). To explore if serelaxin could attenuate GTN tolerance *ex vivo*, mouse aortic rings were co-incubated with GTN (10 μM) and serelaxin (10 nM) for 1 h with a 1 h washout period. This significantly increased (*P* < 0.05) both the sensitivity and maximum relaxation to GTN compared with aortae that were co-incubated with GTN and placebo (**Figure 1B**). However, a lower concentration of serelaxin (3 nM) with 10 μM of GTN did not significantly improved the sensitivity and maximum relaxation to GTN. Incubation of mouse aortic rings with serelaxin (1, 3, and 10 nM) had no effect on the sensitivity and maximum response to subsequent incubation in GTN (**Figure 1C**).

### Serelaxin Attenuates GTN Tolerance and Reduces Superoxide Production in the Rat Aorta *In vivo*

**FIGURE 2:**
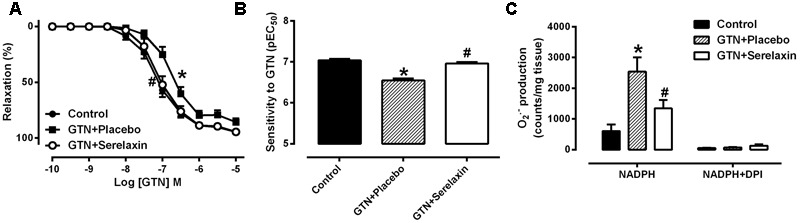
**Concentration-response curve to (A)** GTN and **(B)** sensitivity to GTN in endothelium-intact aorta from control, GTN+placebo or GTN+serelaxin rats for 3 days. **(C)** Aortic superoxide production measured by lucigenin-enhanced chemiluminescence assay from control, GTN+placebo or GTN+serelaxin rats for 3 days. Values are expressed as mean ± SEM, *n* = 8–11 per group. ^∗^*P* < 0.05, significantly different to control, #*P* < 0.05, significantly different to GTN+placebo (one-way ANOVA, Tukey’s test). Maximum relaxation (Rmax) values are shown in **Table 2**.

Mean plasma concentrations of serelaxin in the GTN+serelaxin co-treated rats after 3 days of infusion was 79.2 ± 10.3 ng/mL (range: 51.0–116.3 ng/mL, mean concentration equivalent to ∼13.2 nM, *n* = 8). Serelaxin was not detectable in the plasma of control and GTN+placebo rats. Continuous infusion of low-dose GTN for 3 days resulted in the development of tolerance to GTN, demonstrated by a significant reduction in both the sensitivity (*F*_2,27_ = 5.34, *P* = 0.01) and maximum relaxation (*F*_2,27_ = 5.66, *P* = 0.02) to GTN (**Figures 2A,B** and **Table 2**) in the rat aorta compared with control rats. Conversely, co-treatment of rats with GTN+serelaxin significantly increased the sensitivity (*P* = 0.04) and maximum relaxation (*P =* 0.02) to GTN compared with GTN+placebo, indicating that serelaxin co-treatment prevented GTN tolerance *in vivo*. Superoxide production in the aorta was significantly (*F*_2,28_ = 7.72, *P* = 0.002) increased in the GTN+placebo rats compared with control rats, whereas co-treatment of GTN-infused rats with serelaxin significantly (*P* = 0.04) attenuated superoxide levels (**Figure 2C**).

**Table 2:** A comparison of sensitivity (pEC_50_) and maximum relaxation (R_max_) to GTN, DEA/NO and ACh in endothelium-intact aorta isolated from control, GTN+placebo and GTN+serelaxin treated rats.

	Control		GTN+placebo		GTN+serelaxin	
Vasodilators	pEC_50_	R_max_ (%)	pEC_50_	R_max_ (%)	pEC_50_	R_max_ (%)
GTN	7.04 ± 0.04	94 ± 2	6.55 ± 0.05*	86 ± 3*	6.96 ± 0.04ˆ#	94 ± 1ˆ#
DEA/NO	6.81 ± 0.05	100 ± 1	6.84 ± 0.05	101 ± 1	6.92 ± 0.06	101 ± 1
ACh	6.18 ± 0.04	90 ± 4	6.28 ± 0.04	88 ± 2	6.18 ± 0.03	95 ± 4

*n = 6–11. Results are shown as mean ± SEM. ^∗^*P* < 0.05 versus control, ^#^*P* < 0.05 versus GTN+placebo, one-way ANOVA with Tukey’s *post hoc* analysis.*

### Serelaxin Attenuates the Reduction of ALDH-2 Expression in the Aorta of GTN-Treated Rats

**FIGURE 3:**
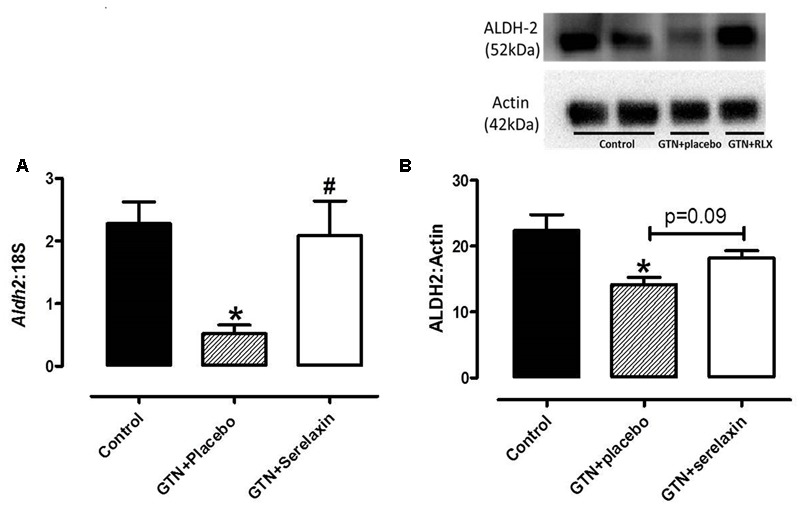
**(A)** Quantitative analysis of *Aldh2* mRNA expression and **(B)** Western blot analysis of ALDH-2 protein expression in the aorta from control, GTN+placebo or GTN+serelaxin rats for 3 days. Values are 2^-ΔCt^ ± SEM, *n* = 8–11 per group. Representative blot of ALDH-2 protein expression is shown above the respective panels, *n* = 5–6 per group. ^∗^ Significantly different to control, # significantly different to GTN+placebo, *P* < 0.05 (one-way ANOVA, Tukey’s *post hoc* test).

Continuous infusion of low-dose GTN for 3 days significantly (*F*_2,27_ = 5.03, *P* = 0.02) reduced both gene (**Figure 3A**) and protein (**Figure 3B**) expression of ALDH-2 in the rat aorta. These negative effects of GTN on *Aldh*-2 gene expression (*P* = 0.03) were prevented in the aorta of rats co-treated with serelaxin for 3 days (**Figure 3A**). A similar effect was observed on ALDH-2 protein expression (**Figure 3B**) but it failed to reach statistical significance (*P* = 0.09).

**FIGURE 4:**
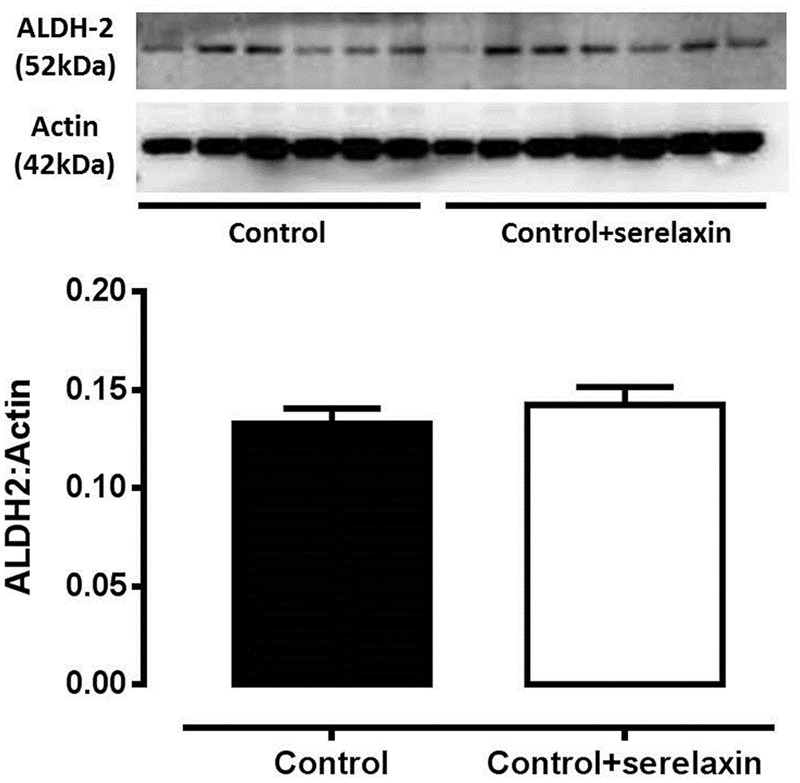
**Western blot analysis of ALDH-2 protein expression in the aorta from control, control+serelaxin rats for 3 days.** Protein extracts used in this experiment were previously published in Leo et al. (2016c). Representative blot of ALDH-2 protein expression is shown above the respective panels, *n* = 6–7 per group.

To address if serelaxin alone had any effect on ALDH-2 expression in the rat aorta, we utilized aortic protein extract of control and control+serelaxin from our recently published work (Leo et al., 2016c), and analyzed ALDH-2 expression. Although direct comparison cannot be made with samples collected from the present GTN study, the data from **Figure 4** suggested that serelaxin treatment alone had no significant effect on ALDH-2 expression in the rat aorta.

### Serelaxin Does Not Improve Endothelial Function and Cross-Tolerance

**FIGURE 5:**
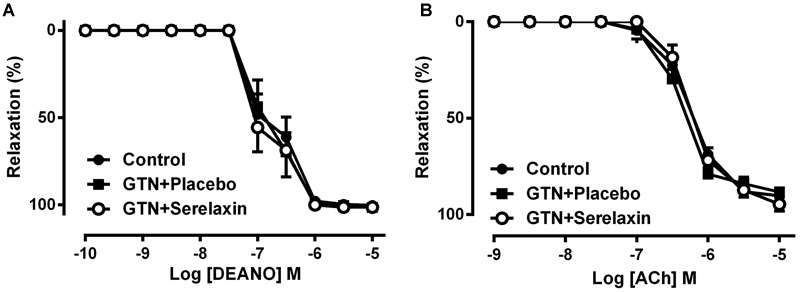
**Concentration-response curve to (A)** DEA/NO and **(B)** ACh in endothelium-intact aorta from control, GTN+placebo or GTN+serelaxin rats for 3 days. Values are expressed as mean ± SEM, *n* = 6–11 per group. pEC_50_ and maximum relaxation (R_max_) values were shown in **Table 2**.

Low-dose GTN infusion for 3 days resulted in comparable responses to the nitrovasodilator, DEA/NO (**Figure 5A** and **Table 2**) or ACh (**Figure 5B** and **Table 2**) in the aorta compared with control, indicating that low-dose GTN treatment had no effect on the development of cross-tolerance or endothelial dysfunction. Similarly, serelaxin co-treatment also had no significant effect on the responses to DEA/NO (**Figure 5A** and **Table 2**) or ACh (**Figure 5B** and **Table 2**) in the aorta. This is consistent with our recently published data (Leo et al., 2016c), showing that serelaxin treatment alone for 3 days did not affect ACh responses (pEC_50_; placebo: 6.24 ± 0.07 vs. serelaxin: 6.38 ± 0.11) in the aorta.

**FIGURE 6:**
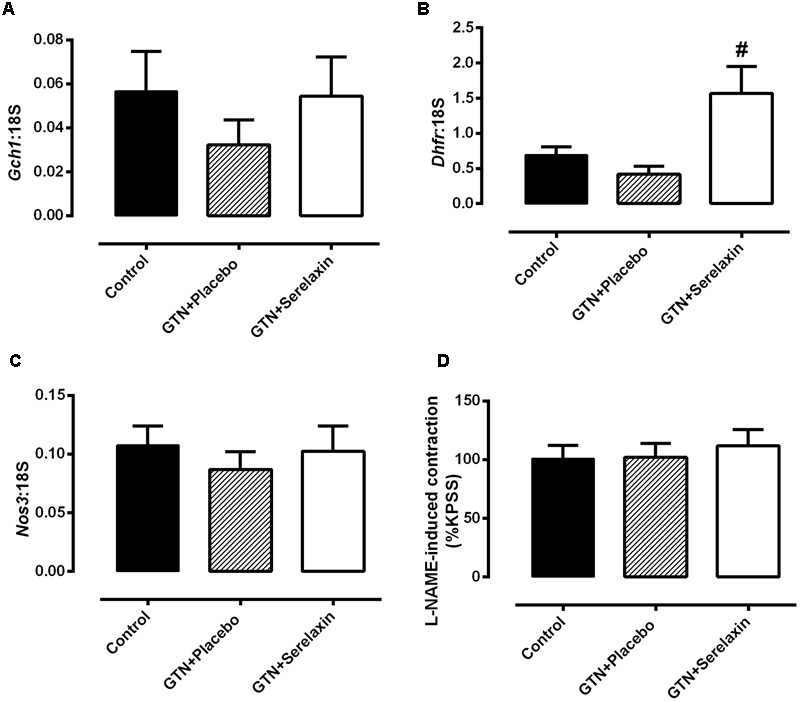
**Quantitative analysis of (A)** Gch1, **(B**) Dhfr, and **(C)** Nos3 mRNA expression in the aorta from control, GTN+placebo or GTN+serelaxin rats for 3 days. **(D)** Basal NOS activity in endothelium-intact aorta from control, GTN+placebo or GTN+serelaxin rats for 3 days. In each group of experiments, aortae were pre-contracted with phenylephrine (PE) to a similar level (∼20% of KPSS contraction) before the addition of L-NAME (200 μmol L^-1^). *n* = 5–8 per group. Values are 2^-ΔCt^ ± SEM. # significantly different to GTN+placebo, *P* < 0.05 (one-way ANOVA, Tukey’s *post hoc* test).

In addition to the lack of endothelial dysfunction, low-dose GTN treatment had no significant effect on mRNA expression of *Nos3* and enzymes that are involved in eNOS uncoupling, GTPCH-1 and DHFR. Furthermore, expression of *Gch1* in the aorta (**Figure 6A**) did not differ significantly in the GTN+serelaxin compared with control or GTN+placebo groups. Similarly, there were no significant effects of serelaxin treatment on *Nos3* (**Figure 6C**) or_L_-NAME-induced contraction (**Figure 6D**), indicating that basal NOS activity was not affected by GTN+placebo or GTN+serelaxin infusion. Of note, although GTN+placebo infusion for 3 days had no effect on *Dhfr* expression compared to control (**Figure 6B**), GTN+serelaxin co-treatment significantly (*P* = 0.04) increased *Dhfr* expression compared to GTN+placebo.

## Discussion

The aims of the present study were to investigate whether or not serelaxin attenuates low-dose GTN tolerance using *ex vivo* and *in vivo* rodent models of GTN tolerance. In the *ex vivo* model, we demonstrated GTN tolerance in the mouse aorta after 1 h of exposure. Co-incubation of GTN and serelaxin reversed GTN tolerance. In the *in vivo* rat model, continuous infusion of low-dose GTN for 3 days reduced the vasodilator response to GTN in the aorta, indicating development of GTN tolerance. This was underpinned by reduced vascular ALDH-2 expression and increased superoxide production. Consistent with the *ex vivo* finding, serelaxin co-infused with low-dose GTN for 3 days enhanced the vasodilator action of GTN and attenuated the development of GTN tolerance. This was accompanied by increased ALDH-2 expression and reduced superoxide production in the aorta.

Glyceryl trinitrate is the most commonly used vasodilator agent for patients with AHF. Unfortunately, the therapeutic efficacy and vasodilator properties of GTN are rapidly blunted with prolonged treatment due to the development of nitrate tolerance (Gori et al., 2001; Münzel et al., 2005, 2011; Schulz et al., 2011). Serelaxin has emerged as a novel vasoprotective peptide (Leo et al., 2016b) with beneficial effects in AHF patients, who were also administered with low dose nitrates (less than 100 μg/kg/h) as standard care (Teichman et al., 2010; Teerlink et al., 2013). In our *ex vivo* study, we provided evidence that serelaxin reverses nitrate tolerance induced by prolonged exposure to GTN in the aorta. Previous studies demonstrated that serelaxin directly activates the sGC-cGMP dependent pathway in adult and fetal vascular smooth muscle cells (Bani et al., 1998; Failli et al., 2005; Sarwar et al., 2015, 2016) and more recently in the kidneys (Wetzl et al., 2016). This suggests that serelaxin could reverse GTN tolerance by increasing the activity of sGC, which will enhance the vasodilator action of nitrates. In the current study, we showed that incubation of increasing concentrations of serelaxin *ex vivo* had no effect on the GTN vasodilator response in the mouse aorta, implying that serelaxin does not interfere with the vasodilator capacity of GTN. Therefore, it is unlikely that serelaxin enhances the sGC activity to augment GTN vasodilator responses. Nonetheless, the *ex vivo* experiment provided “proof of concept” evidence that serelaxin has the ability to attenuate GTN tolerance. Another possible mechanism of serelaxin action is through GTN biotransformation.

Impaired biotransformation of GTN is recognized as a critical factor in the development of GTN tolerance, which is underpinned by reduced expression and activity of ALDH-2 in both the *ex vivo* and *in vivo* setting (Sydow et al., 2004; Hink et al., 2007). To test the hypothesis that serelaxin increases GTN biotransformation, we used an *in vivo* model of low-dose GTN tolerance. This low-dose GTN resulted in a diminished vasodilator response to GTN, accompanied by a reduction in ALDH-2 expression in the rat aorta, consistent with many other studies that used relatively high doses of GTN. More importantly, we showed that serelaxin co-infusion increased ALDH-2 expression, decreased superoxide generation and improved GTN-induced relaxation in the rat aorta. Due to the limited availability of the tissue, we are unable to measure ALDH-2 activity which is a more appropriate than evaluation of protein/gene expression. Reduction of oxidative stress with antioxidant treatments increases ALDH-2 activity and restores GTN vasodilator capacity, providing further evidence that ROS is an important contributing factor in the development of nitrate tolerance (Sydow et al., 2004; Hink et al., 2007).

Serelaxin attenuates several markers of oxidative stress in a number of disease settings (Dschietzig et al., 2012; Collino et al., 2013; Sasser et al., 2014). Specifically, *ex vivo* serelaxin treatment reduces tumor necrosis factor-α stimulated superoxide and nitrotyrosine formation in the rat aorta (Dschietzig et al., 2012). Furthermore, serelaxin significantly decreases free radical species associated with renal ischemia reperfusion injury, while upregulating the endogenous antioxidant enzymes manganese and copper zinc SODs (Collino et al., 2013). Thus, it is logical to hypothesize that serelaxin may reduce ROS levels in the setting of GTN tolerance. Indeed, our data demonstrated that GTN treatment significantly increased superoxide production in the rat aorta, and importantly, serelaxin treatment reduced this. Taken together, we suggest that serelaxin reduces vascular oxidative stress, resulting in enhanced ALDH-2 and GTN-induced relaxation in the rat aorta.

An alternative potential mechanism in which ALDH-2 expression and activity may be modulated by serelaxin is through the PI3K/Akt-dependent signaling pathway. Phosphorylation of Akt increases the expression of ALDH-2 in the heart and human lung epithelial cells, which can be inhibited by the PI3K inhibitor, wortmannin (Xu et al., 2006; Yu et al., 2014). This suggests that ALDH-2 expression is regulated by PI3K/Akt-dependent signaling pathway. The receptors for relaxin, RXFP1 are localized to the vascular smooth muscle cells in the rat aorta (Jelinic et al., 2014). Activation of RXFP1 stimulates the PI3K/Akt-dependent signaling pathway (Conrad, 2010; McGuane et al., 2011b), leading to NO-dependent effects, including vasodilation (Leo et al., 2014b). Thus, we propose RXFP1 activation in the vascular smooth muscle cells by serelaxin stimulates PI3K-dependent phosphorylation of Akt, and increase the expression of cytosolic ALDH-2 expression (Beretta et al., 2012). As a result, biotransformation of GTN is enhanced, preventing the development of GTN tolerance.

A consequence of GTN tolerance is endothelial dysfunction and cross-tolerance to other nitrovasodilators. However, there is considerable variation between studies on the effective duration of GTN treatment required to induce endothelial dysfunction and cross-tolerance to other nitrovasodilators. Endothelial dysfunction, cross-tolerance to NO donors and impaired endothelium-derived NO were observed in rabbit aorta after 3 days of chronic GTN treatment (Münzel et al., 1995). Similarly, 3 days of GTN infusion caused a small but significant reduction in ACh-mediated relaxation in the rat aorta, but there was no cross-tolerance to DEA/NO (Irvine et al., 2011). Six days of chronic nitrate treatment also led to decreased ACh-mediated blood flow in the forearm of healthy human subjects (Gori et al., 2001). In our study, despite increased vascular oxidative stress, we were unable to achieve endothelial dysfunction in the rat aorta or cross tolerance in response to 3 days of GTN infusion *in vivo*, as determined by comparable relaxation responses to ACh and DEA/NO. Furthermore, there were no changes to basal NOS activity (determined pharmacologically) or *enos, Gtpch1* and *Dhfr* gene expression. This phenomenon has been reported in other studies; for example endothelial function is preserved despite increased superoxide production in diabetic carotid arteries (Leo et al., 2010) and the aorta of relaxin-deficient mice (Ng et al., 2015). This may be due to compensatory changes in the mechanisms of endothelium-dependent relaxation in response to increased oxidative stress (Leo et al., 2010; Ng et al., 2015). It is also possible that we did not observe endothelial dysfunction because our study used a clinically relevant, low-dose of GTN which is at least 30-fold lower than other studies (Irvine et al., 2011), which reported endothelial dysfunction but no cross tolerance to DEA/NO in the rat aorta. Similar to Münzel and colleagues (Daiber et al., 2004) who used relatively higher doses of GTN, we also successfully established an animal model with GTN tolerance, underpinned by reduced ALDH-2 expression and increased oxidative stress. More importantly, in this animal model, we demonstrated that serelaxin treatment prevented the development of GTN tolerance which was the primary objective of the present study. We also suggest that the development of nitrate tolerance is an early event and precedes endothelial dysfunction and/or cross-tolerance in the rat aorta.

Serelaxin treatment in rats enhances endothelium-dependent relaxation in vascular-region dependent manner. This is achieved through activation of various endothelium-derived vasodilators including NO and PGI_2_ (Jelinic et al., 2014; Leo et al., 2014b, 2016c). Specifically, in the rat aorta, the increase in NO is achieved through increased phosphorylation of eNOS after 2 days but not 3 days of serelaxin treatment. Consistent with previously reported findings (Leo et al., 2016c), serelaxin treatment for 3 days had no effect on either basal NOS activity, or ACh-mediated relaxation in the aorta. Despite the lack of functional changes in endothelium-derived NO-dependent relaxation, serelaxin caused a significant increase in *Dhfr* expression. Thus it appears that serelaxin may be influencing the signaling machinery of the eNOS pathway, perhaps by regulating DHFR.

## Conclusion

Our study demonstrates that serelaxin attenuates the development of GTN tolerance in the rat and mouse aorta. More importantly, serelaxin’s ability to reverse nitrate tolerance is achieved by decreasing superoxide production and increasing ALDH-2 expression. We suggest that co-administration of serelaxin has the potential to improve nitrate efficacy clinically for AHF patients.

## Author Contributions

CL and DF wrote the manuscript. All authors (CL, DF, LT, HN, SM, and LP) contributed to the design of the work; or the acquisition, analysis, or interpretation of data for the work; critically revised the work for important intellectual content; agreed to be accountable for all aspects, including the accuracy or integrity of the work; and approved the final version of the work to be published.

## Conflict of Interest Statement

The authors disclose that this project was partially funded by Novartis Pharma AG, who also provided the serelaxin as a condition of an Australian Research Council Linkage Grant. Professor LP was also a paid consultant for Novartis Pharma AG.

## Abbreviations

ACh: acetylcholine
ALDH-2: aldehyde dehydrogenase-2
cGMP: cyclic guanosine monophosphate
COX: cyclooxygenase
DHFR: dihydrofolate reductase
EDH: endothelium-derived hyperpolarization
eNOS: endothelial nitric oxide synthase
GTN: glyceryl trinitrate
GTPCH: GTP cyclohydrolase-1
IK_Ca_: intermediate-conductance calcium-activated K^+^ channel
iNOS: inducible nitric oxide synthase
L-NAME: *Nω*-nitro-L-arginine methyl ester hydrochloride
NO: nitric oxide
PGI_2_: prostacyclin
ROS: reactive oxygen species
RXFP1: relaxin/insulin-like family peptide receptor 1
